# ITALIAN TRANSLATION AND CULTURAL ADAPTATION OF THE AGITATED BEHAVIOR SCALE (ABS-I) IN PATIENTS WITH ACQUIRED BRAIN INJURIES

**DOI:** 10.2340/jrm.v56.11663

**Published:** 2024-04-04

**Authors:** Chiara-Camilla DERCHI, Pietro ARCURI, Angela COMANDUCCI, Antonio CARONNI, Chiara PAGLIARI, Alessandro VIGANÒ, Eleonora VOLPATO, Jorge NAVARRO, Pietro Davide TRIMARCHI

**Affiliations:** 1 IRCCS Fondazione Don Carlo Gnocchi ONLUS, Milan, Italy; 2 Università Campus Bio-Medico di Roma, Rome, Italy; 3 Department of Neurorehabilitation Sciences, IRCCS Istituto Auxologico Italiano, Ospedale San Luca, Milan, Italy; 4 Department of Biomedical Sciences for Health, University of Milan, Milan, Italy; 5 Department of Psychology, Università Cattolica del Sacro Cuore, Milan, Italy

**Keywords:** Agitation Behavior Scale, cross-cultural adaptation, acquired brain injuries, PTA, PTCS, rehabilitation

## Abstract

**Objective:**

The objective of this study was to produce a cross-cultural adaptation in Italian of the Agitated Behavior Scale (ABS), originally developed in English, as the first of two stages that also include cross-cultural validation and allow a clinical scale to be used in the proper setting such as rehabilitation units.

**Methods:**

In order to adapt the ABS scale to a different cultural environment, five consecutive steps were performed: (1) forward translations (*n* = 8), (2) synthesis of the 8 forward translations to obtain a first shared italian version (ABS_I_trial), (3) back translations (*n* = 3), (4) creation of an expert committee to evaluate forward and back translations and finally (5) the cognitive debriefing.

**Results:**

After the five steps, including forward translations and back translations, the process of committee verification and judgement and the evaluative step of cognitive debriefing, high comprehensibility of all items was found, resulting in an Italian translation version of ABS suitable for application in a clinical setting.

**Conclusion:**

ABS translation was produced by means of a standardized procedure aimed at minimizing cross-cultural gaps. The expert committee evaluated the version produced as highly understandable in Italian. Further steps, such as the subsequent validation of its psychometric properties, are needed to employ this translation in a clinical setting.

LAY ABSTRACT

The objective of this study was to produce an Italian adaptation of the Agitated Behavior Scale, a clinical tool useful to assess, in a standardized way, the level and quality of agitation of patients suffering from traumatic brain injury during the acute phase of recovery. A formal 5-step procedure following the existing recommendations and guidelines was defined and implemented. Following the completion of five stages, encompassing forward and backward translations, committee verification and judgment, and the evaluative process of cognitive debriefing, it was determined that all items exhibited high comprehensibility. The majority of items attained a median score of 5, signifying the highest rating on the 5-point Likert scale utilized for evaluation of items' clarity. Consequently, an Italian translated version of the Agitated Behavior Scale was deemed suitable for implementation in clinical environments. Due to the high relevance of the management of agitated behaviour in patients suffering traumatic brain injury, the resulting Italian version of the Agitated Behavior Scale could fill a gap in the Italian rehabilitation setting, due to a lack of adequately adapted tools for the assessment of post-traumatic agitation.

The Agitated Behavior Scale (ABS [1]) was primarily developed to evaluate, in a standardized way, the level and quality of agitation of patients suffering from traumatic brain injury (TBI) during the acute phase of recovery (2). Its main objective is to assess and longitudinally monitor the agitation of patients in order to tailor clinical interventions. This scale is also currently included in the Confusion Assessment Protocol (CAP [3]), recently developed to properly frame the core clinical features of a condition known as post-traumatic confusional state (PTCS [4]). In addition to this, several studies have also demonstrated the effectiveness of the ABS when applied to assess a population of patients different from those with acquired brain injury, such as patients with progressive dementia primarily due to Alzheimer’s disease (5) or patients with severe mental disorders (6). Moreover, robust psychometric data concerning ABS are available both in its original English version (7) and in other languages (6), showing that this scale is a reliable instrument for measuring agitation.

The accurate assessment of agitation in the clinical context is a crucial first step for the definition of possible interventions to manage this challenging patient behaviour. Despite the widespread use of ABS, to the best of our knowledge, no validated Italian translations are currently available. The aim of the present study is to develop an Italian version of the ABS (ABS-I) as the first step of the cross-cultural adaptation process (8).

## METHODS

**Fig. 1:**
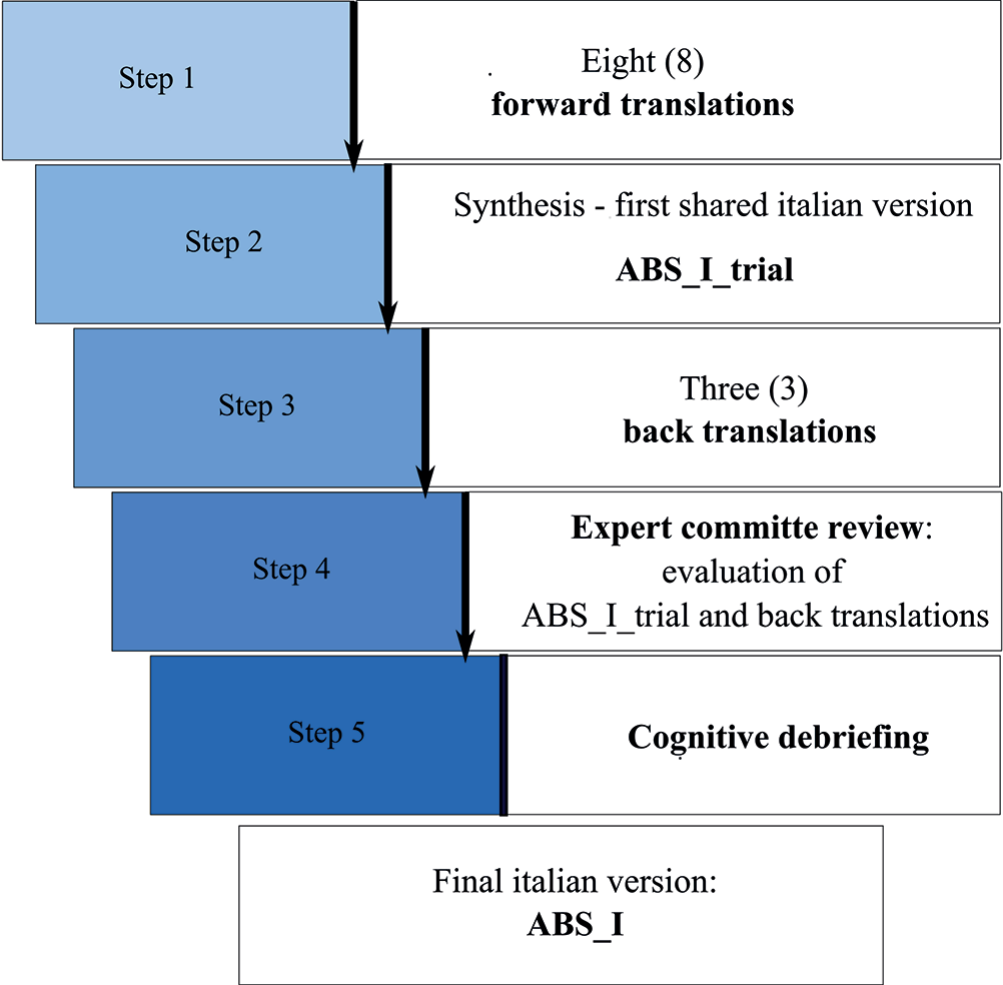
Flow of the procedures for the Italian version of the Agitated Behavior Scale (ABS-I).

A formal 5-step process (Fig. 1) following the existing recommendations and guidelines (ISPOR principles of good practice [9] for the general approach and the German cross-cultural version of the English ABS [10], for a specific example of translation and adaptation in a European language) was defined and implemented to develop the Italian version of the ABS. The multi-step approach recommends a sufficient number of forward and back translations from the original version in order to minimize ambiguities and discrepancies in the interpretation of the original items. For this purpose, as a first step we produced 8 forward translations. The ABS English version (1) was used as the questionnaire source version. As the ABS could be used by different specialists involved in the clinical team (such as medical doctor, neuropsychologist, researcher) we selected 4 Italian mother-tongue translators with an English proficiency level between B2 and C1 of the Common European Framework of Reference for Languages (CEFRL) who are aware of the objective of the study by an interdisciplinary team (1 neuropsychologist, 1 researcher and 2 medical doctors) and 4 *naif* Italian mother-tongue translators, with the same level of proficiency in English, who are not involved in the clinical management of patients with acquired brain injury (3 medical doctors and 1 researcher). As a second step after the production of the translations, we synthesized all the discrepancies and differences with the group of 8 translators, producing a first shared Italian version (ABS_I_trial). In the third step, the ABS_I_trial was back translated into English by 3 back translators. Back translators were selected both on the basis of an optimal command of English (C2 level of the CEFRL) and on knowledge of the clinical setting in which ABS is usually employed: 1 back translator was bilingual and an expert in the field of interest (medical doctor and PhD); the second back translator was an Italian mother-tongue professional translator from English to Italian and vice versa; and the third was an Italian mother-tongue senior clinical trial manager. As 2 of the 3 back translators were not English mother tongue, the 3 back translations were submitted to 1 of the authors of the original ABS version for her evaluation. In step 4 a committee of 5 experts (1 medical doctor, 2 researchers, 1 professional translator and 1 clinical trial manager) with excellent proficiency both in languages and in the field of interest was created in order to evaluate and compare the ABS_I_trial and the 3 back translations. With the objective of standardizing the process of analysis of the different translations by the committee, an evaluation grid was proposed. The committee has to evaluate each translation on the basis of semantic and syntactic properties of texts and contextual properties (i.e. how well the produced text fits the context of use, that is, the evaluation of its pragmatic properties). Finally, as a fifth step the adapted version obtained of the ABS scale underwent a cognitive debriefing, during which 8 psychologists, 3 medical doctors and 1 physiotherapist evaluated the clarity and the comprehensibility of each part of the ABS scale (instructions, *n* = 1; items, *n* = 14; and the overall scale, *n* = 1) of the final translation by a 5-point Likert scale (1 = inadequate, 5 = very good). For the results obtained we then computed the median and the interquartile range (IQR). The cognitive debriefing step has been implemented in order to quantitatively assess the level of clarity and comprehensibility of the Italian version obtained of the ABS.

## RESULTS

As regards the first phase (steps 1 and 2), the production of 8 different forward translations converged into 1 translation (ABS_I_trial) after a group brainstorming to solve the linguistic issues due to adaptation in the specific clinical context. Agreement on a single translation was reached by finding the most suitable terms in the clinical setting of application, namely in patients with agitation due to traumatic brain injury.

In terms of the second stage (step 3), where the aim of the 3 back translations was to bridge the gap between the original English version and the Italian translation, it emerged that the 3 back translations showed good similarity to the original English version, a result further validated by one of the authors of the original version of the ABS

The final phase (steps 4 and 5) was based on the comparison of forward and back translations by a committee of experts. The committee’s evaluation showed very good consistency between the different translations produced and the original version. The committee highlighted only some stylistic changes to improve the readability of the scale. The results of cognitive debriefing showed that both instructions and each item of the scale have been judged as highly clear and comprehensible by the expert involved in this evaluation phase. As indicated in Table I, almost all items had a median value of 5, which corresponds to the highest value of the 5-point Likert scale used during assessment.

**Table I:** Median and interquartile range (IQR) for instructions, each item and the overall scale as judged by participants at cognitive debriefing.

Item + instructions: Overall scale	Median (IQR)
Instructions	5 (1)
Item 1	5 (0.75)
Item 2	5 (1)
Item 3	5 (1)
Item 4	5 (1)
Item 5	5 (0)
Item 6	5 (0.75)
Item 7	5 (1)
Item 8	4 (1)
Item 9	5 (0)
Item 10	5 (1)
Item 11	5 (0.75)
Item 12	5 (0)
Item 13	5 (0.75)
Item 14	5 (0.75)
Overall scale judgement	5 (1)

## DISCUSSION

Following an acute acquired brain injury due to trauma, patients might experience a period of recovery where they are responsive but confused, known as PTCS (3, 4). Confusion and/or agitation are common during emergence from a disorder of consciousness (i.e. minimally conscious state) and are characterized by a variety of symptoms including attentional and memory impairment, spatio-temporal disorientation, fluctuations, sleep disturbance, decreased arousal, psychosis and agitation.

The Agitated Behavior Scale is proven as a reliable instrument for measuring agitation in patients with traumatic brain injury (7, 11) and has been included in the CAP to diagnose and monitor a PTCS.

To the best of our knowledge, this is the first Italian adaptation of the ABS. After all evaluation steps , the translation obtained and cultural adaptation showed very good comprehensibility and clarity as judged by a group of experts working in intensive and long-term rehabilitation settings. No specific language difficulties emerged during the several steps followed, and the original English expressions of the ABS did not need to be modified in the process of cultural adaptation. This is in line with others’ experiences of adaptation in European languages (e.g. Spanish translation [6]) and maybe suggests that the phenomenology of behaviours recollected during the application of the ABS is quite typical and clearly observable in clinical settings, and therefore also simple to describe in language form and independent of sociocultural differences. The resulting Italian version of the ABS could fill a gap in the Italian rehabilitation setting, due to a lack of adequately adapted tools for the assessment of post-traumatic agitation.

### Limitations

This study has suffered from some limitations, the principal one being that psychometric properties of the ABS-I have not been tested and this will be a crucial step for its utilization in clinical settings. A further limitation relates to the unavailability of training materials in Italian, so a future step to be implemented will be to translate these in order to promote the correct use of the scale.

### Conclusion

This study of adaptation of the ABS to the Italian language provided the first agitation assessment for use in intensive and long-term rehabilitation for patients with TBI. This is the product of translation and cultural adaptation process following recommended guidelines. Further steps, on a multicentric basis, are needed in order to validate the psychometric properties of the ABS-I and apply it as a validated instrument in the clinical setting.
